# Supplementary material for: Stimulation strength controls the rate of initiation but not the molecular organisation of TCR-induced signalling
Source: eLife. 2020 May 15;9:e53948. doi: 10.7554/eLife.53948 (PMC7308083; doi:10.7554/eLife.53948)
Supplement: Transparent reporting form [file elife-53948-transrepform.docx]

***eLife’s* transparent reporting form**

We encourage authors to provide detailed information *within their submission* to facilitate the interpretation and replication of experiments. Authors can upload supporting documentation to indicate the use of appropriate reporting guidelines for health-related research (see [EQUATOR Network](http://www.equator-network.org/%20)), life science research (see the [BioSharing Information Resource](https://biosharing.org/)), or the [ARRIVE guidelines](http://www.plosbiology.org/article/info:doi/10.1371/journal.pbio.1000412) for reporting work involving animal research. Where applicable, authors should refer to any relevant reporting standards documents in this form.

If you have any questions, please consult our Journal Policies and/or contact us: [editorial@elifesciences.org](mailto:editorial@elifesciences.org).

**Sample-size estimation**

- You should state whether an appropriate sample size was computed when the study was being designed
- You should state the statistical method of sample size computation and any required assumptions
- If no explicit power analysis was used, you should describe how you decided what sample (replicate) size (number) to use

Please outline where this information can be found within the submission (e.g., sections or figure legends), or explain why this information doesn’t apply to your submission:

No sample size calculation was performed prior to data collection. Our experiments examined the distributions of cell through single-cell analyses. The sample sizes used provided adequate information about these distributions as demonstrated through biological reproducibility.

**Replicates**

- You should report how often each experiment was performed
- You should include a definition of biological versus technical replication
- The data obtained should be provided and sufficient information should be provided to indicate the number of independent biological and/or technical replicates
- If you encountered any outliers, you should describe how these were handled
- Criteria for exclusion/inclusion of data should be clearly stated
- High-throughput sequence data should be uploaded before submission, with a private link for reviewers provided (these are available from both GEO and ArrayExpress)

Please outline where this information can be found within the submission (e.g., sections or figure legends), or explain why this information doesn’t apply to your submission:

Findings were reproduced through biological replicates and complementary measurements using other techniques. A biological replicate is defined as one using cells from an independent mouse or pool of mice, while an independent experiment is defined as an experiment that took place on an entirely different day with independent mice. The number of biological replicates and independent experiments are indicated for each figure in the figure legends of the manuscript as well as Supplementary File 1.

Experiments were only deemed as failures and thereby excluded if technical experimental errors or equipment malfunction made data unreliable.

Individual samples were only excluded from one mass cytometry experiment when an error in barcoding made data unusable. The CD69-143Nd antibody was excluded from the analyses in mass cytometry experiment 1 and excluded from the staining panel in experiment 2 as detailed in the methods due to lack of signal in the staining conditions used. Samples were not excluded from flow cytometry analyses.

**Statistical reporting**

- Statistical analysis methods should be described and justified
- Raw data should be presented in figures whenever informative to do so (typically when N per group is less than 10)
- For each experiment, you should identify the statistical tests used, exact values of N, definitions of center, methods of multiple test correction, and dispersion and precision measures (e.g., mean, median, SD, SEM, confidence intervals; and, for the major substantive results, a measure of effect size (e.g., Pearson's r, Cohen's d)
- Report exact p-values wherever possible alongside the summary statistics and 95% confidence intervals. These should be reported for all key questions and not only when the p-value is less than 0.05.

Please outline where this information can be found within the submission (e.g., sections or figure legends), or explain why this information doesn’t apply to your submission:

Information on statistical analysis is described within the figure legends and methods. Raw data is presented in the figures as individual points where indicated. Summary statistics from mass cytometry analyses are included as Supplementary File 5.

(For large datasets, or papers with a very large number of statistical tests, you may upload a single table file with tests, Ns, etc., with reference to sections in the manuscript.)

**Group allocation**

- Indicate how samples were allocated into experimental groups (in the case of clinical studies, please specify allocation to treatment method); if randomization was used, please also state if restricted randomization was applied
- Indicate if masking was used during group allocation, data collection and/or data analysis

Please outline where this information can be found within the submission (e.g., sections or figure legends), or explain why this information doesn’t apply to your submission:

Experimental groups consisted of cells treated under different stimulatory conditions. In all experiments, cells for each condition were taken from a homogeneous pool, which contained cells from 1 to 3 mouse spleens (see Methods). For mass cytometry experiments, cells from different stimulatory conditions were barcoded and pooled as detailed in Supplementary File 1 for staining and analyses to avoid confounding technical effects. All analyses were performed without considering the identity of sample groups except where a control sample was necessarily used as reference as described in the methods.

**Additional data files (“source data”)**

- We encourage you to upload relevant additional data files, such as numerical data that are represented as a graph in a figure, or as a summary table
- Where provided, these should be in the most useful format, and they can be uploaded as “Source data” files linked to a main figure or table
- Include model definition files including the full list of parameters used
- Include code used for data analysis (e.g., R, MatLab)
- Avoid stating that data files are “available upon request”

Please indicate the figures or tables for which source data files have been provided:

Raw mass cytometry data can be found on the Flow Repository, accession numbers FR-FCM-Z2CX and FR-FCM-Z2CP.

Full results of mass cytometry analyses are included as Supplementary File 5.

Source data for summary plots of flow cytometry-measured signaling markers in T cells stimulated with peptide-loaded BMDCs (Figure 7a) are included as Figure 7 – Source Data File 1.

Analysis code is available at

<https://github.com/MarioniLab/SignallingMassCytoStimStrength>
